# Probabilistic computation underlying sequence learning in a spiking attractor memory network

**DOI:** 10.1186/1471-2202-14-S1-P236

**Published:** 2013-07-08

**Authors:** Philip Tully, Henrik Lindén, Matthias H Hennig, Anders Lansner

**Affiliations:** 1 Department of Computational Biology, Royal Institute of Technology (KTH), S-10044, Stockholm, Sweden; 2 Department of Numerical Analysis and Computing Science, Stockholm University, S-10044, Stockholm, Sweden; 3 Stockholm Brain Institute, Karolinska Institutet, 171 77, Stockholm, Sweden; 4 Institute for Adaptive and Neural Computation, University of Edinburgh, EH8 9AB, Edinburgh, UK

## 

Many cognitive and motor functions are enabled by the temporal representation and processing of stimuli but it remains an open issue how neuronal circuits could reliably encode such sequences of information. We consider the task of generating and learning spatiotemporal spike patterns in the context of an attractor memory network, in which each memory is stored in a distributed fashion represented by increased firing in pools of excitatory neurons. Excitatory activity is locally modulated by inhibitory neurons representing lateral inhibition that generates a type of winner-take-all dynamics. Networks of this type have previously been shown to exhibit switching between a non-coding ground state and low-rate memory state activations displaying gamma oscillations [1]; however, stable sequential associations between different attractors were not present.

Assuming a probabilistic framework in which local neuron populations discretely encode uncertainty about an attribute in the external world (e.g. a column in visual cortex tuned to a specific edge orientation), we model inter-module synapses using the Bayesian Confidence Propagation Neural Network (BCPNN) plasticity rule [2]. We use a spike-based version of BCPNN in which synaptic weights are statistically inferred by estimating the posterior likelihood of activation for the postsynaptic cell upon presentation of evidence in the form of presynaptic activity patterns. Probabilities are estimated on-line using local exponentially weighted moving averages, with time scales that are biologically motivated by the cascade of events involved in the induction and maintenance of long-term plasticity. Modulating the kinetics of these traces is shown to shape the width of the STDP kernel, which in turn allows attractors to be learned forwards or backwards through time. Stable learning is confirmed by aunimodal stationary weight distribution. Inference additionally requires modification of a distinct neuronal component, which we interpret as a correlate of intrinsic excitability. Such synaptic [3] and nonsynaptic [4] mechanisms were specifically shown to be relevant for learning and inference. In broader terms, our model instead suggests the presence of and interaction between all of these processes in approximating Bayesian computation.

Introducing plastic BCPNN synaptic projections into the attractor network model allows for stable associations between distinct network states. Associations are mediated by different synaptic timescales [5] with fast (AMPA type) and slower (NMDA type) dynamics that in conjunction with the spiking BCPNN rule produce sequences of attractor activations. We demonstrate the feasibility of our model using network simulations of integrate-and-fire neurons, and find that the ability to learn sequences depends on the specific structure of the inhibitory microcircuitry and on the local balance of excitation and inhibition in the network. Preliminary results show that the network can reliably store spatiotemporal patterns consisting of hundreds of discrete network states using just a few thousand neurons. Moreover, excitatory pools can participate multiple times in the sequence, suggesting that spiking attractor networks of this type could support an efficient combinatorial code. Our model provides novel insights into how local and global computations found throughout neocortex and hippocampus, framed in the context of probabilistic inference, could contribute to generating and learning sequential neural activity.

## References

[B1] Lundqvist M Compte A Lansner A Bistable, irregular firing and population oscillations in a modular attractor memory network PLoS Comput Biol 2010 6 6 e1000803 10.1371/journal.pcbi.1000803 20532199 PMC2880555

[B2] Lansner A Ekeberg Ö A One-Layer feedback artificial neural network with a Bayesian learning rule Int J Neural Syst 1989 1 77 87 10.1142/S0129065789000499

[B3] Keck C Savin S Lücke J Feedforward inhibition and synaptic scaling - two sides of the same coin? PLoS Comput Biol 2012 8 3 e1002432 10.1371/journal.pcbi.1002432 22457610 PMC3310709

[B4] Habenschuss S Bill J Nessler B Homeostatic plasticity in Bayesian spiking networks as Expectation Maximization with posterior constraints Adv Neural Inf Process Syst 2012 25 782 790

[B5] Kleinfeld D Sequential state generation by model neural networks Proc Natl Acad Sci USA 1986 83 24 9469 9473 10.1073/pnas.83.24.9469 3467316 PMC387161

